# Comparative Study of Different Diagnostic Routine Methods for the Identification of *Acinetobacter radioresistens*

**DOI:** 10.3390/microorganisms10091767

**Published:** 2022-08-31

**Authors:** Richard Bigge, Boyke Bunk, Wolfram W. Rudolph, Florian Gunzer, Sina M. Coldewey, Thomas Riedel, Percy Schröttner

**Affiliations:** 1 Institute for Microbiology and Virology, University Hospital Carl Gustav Carus, 01307 Dresden, Germany; 2 Leibniz Institute DSMZ-German Collection of Microorganisms and Cell Cultures GmbH, 38124 Braunschweig, Germany; 3 Department of Hospital Infection Control, University Hospital Carl Gustav Carus, 01307 Dresden, Germany; 4 Department of Anesthesiology and Intensive Care Medicine, Jena University Hospital, 07745 Jena, Germany; 5 Septomics Research Center, Jena University Hospital, 07747 Jena, Germany; 6 Center for Sepsis Control and Care, Jena University Hospital, 07747 Jena, Germany; 7 German Center for Infection Research (DZIF), Partner Site Hannover-Braunschweig, 38124 Braunschweig, Germany

**Keywords:** *Acinetobacter radioresistens*, VITEK 2, MALDI-TOF MS, 16S rRNA gene, *rpoB*, average nucleotide identity, digital DNA-DNA hybridization

## Abstract

Recent publications indicate that *A. radioresistens* can cause infections in humans, even though it is rarely reported in routine diagnostics. However, the fact that it is infrequently detected may be explained by the misidentification of the species by conventional methods. It is also likely that *A. radioresistens* is not considered clinically relevant and therefore not consistently included in diagnostic results. To elucidate the medical significance of this probably clinically underestimated bacterial species, we created a well-documented reference strain collection of 21 strains collected in routine diagnostics. For further analysis of *A. radioresistens*, it is essential to know which methods can be used to achieve a trustworthy identification. We, therefore, compared three methods widely used in routine diagnostics (MALDI-TOF MS, VITEK 2, and sequencing of housekeeping genes) in terms of secure and reliable identification of *A. radioresistens*. As reference methods, whole genome-based approaches were applied. VITEK 2 led to misidentification for four strains. However, MALDI-TOF MS and sequencing of housekeeping genes led to reliable and robust identifications.

## 1. Introduction

The species *Acinetobacter radioresistens* was first described by Nishimura et al. in 1988 as a non-fermenting, Gram-negative, and rod-shaped bacterium, which forms white to yellow tinted colonies that are opaque, round, and convex [1]. These bacteria can utilize various carbon sources [1]. The reason the species was named “radioresistens” is owed to the fact that the first isolates (including the type strain) were collected from sterilized cotton in the course of an experiment aimed to evaluate the effectiveness of γ-ray sterilization using a cobalt-60 source. The tested species turned out to be resistant to γ-rays [1,2]. In addition, *A. radioresistens* withstands extreme environmental conditions such as desiccation, UV radiation or vapor, and plasma phase H_2_O_2_ [3].

*A. radioresistens* has long been considered a non-pathogenic member of the human skin flora [4]. More recent reports, however, clearly underline that *A. radioresistens,* indeed, is pathogenic for humans. The first case supporting this assumption was published by Visca et al. in 2001. The authors reported on bacteremia in an HIV-positive patient, which was caused by *A. radioresistens* [5]. To date, several other cases (one even with a fatal outcome) have been published, underlining the pathogenic potential of *A. radioresistens* [6,7,8,9]. Furthermore, in 2008, Poirel et al. detected the carbapenemase gene *bla_OXA-23_* within *A. radioresistens* [10]. The corresponding enzyme is currently widely distributed amongst various species of the genus *Acinetobacter* including the members of the *A. baumannii* complex. Therefore, mechanisms have been suggested that support the transfer of these ß-lactamase genes among the different species of the genus via transposable elements or insertion sequences [11,12].

These facts, therefore, suggest that a more detailed examination of *A. radioresistens* is warranted and useful concerning its clinical significance. However, an indispensable prerequisite to start such an investigation is the availability of a well-characterized strain collection of isolates derived from routine diagnostics that serve as reference isolates. To set up such a collection, it is essential to know which routine method of bacterial identification is the most suitable for generating accurate results [13]. We therefore compared different methods for bacterial identification commonly used in microbiological routine laboratories (MALDI-TOF MS, VITEK 2, and sequencing of housekeeping genes such as the 16S rRNA gene). In addition, the extent to which the sequencing of *rpoB* is suitable for diagnostic purposes was investigated. The whole-genome-based applications “Average Nucleotide Identity (ANI)” and “digital DNA-DNA Hybridization (dDDH)” (currently regarded as molecular gold-standard for bacterial species identification) were chosen as reference methods in this study [14,15].

## 2. Materials and Methods

### 2.1. Collection of A. radioresistens Reference Strains

Between 2013 and 2021, a total of 16 isolates were collected from routine clinical diagnostics at the Institute of Medical Microbiology and Virology, University Hospital Carl Gustav Carus Dresden (Dresden, Germany). The bacteria were stored in a Pro-Lab diagnostics Microbank^TM^ (Fisher Scientific, Schwerte, Germany). Before starting the investigations, the bacteria were additionally deposited at the Leibniz Institute DSMZ-German Collection of Microorganisms and Cell Cultures (Braunschweig, Germany). Only previously curated strains were used for the experiments (Table 1).

To add a wider range of intraspecies biodiversity to the collection, other researchers were asked to provide isolates for our study. In total, five isolates from other research groups (located in the USA, Switzerland, and Norway) could be included in our investigations (Table 2).

In addition, the *A. radioresistens* type strain DSM 6976^T^ was purchased from the DSMZ (Braunschweig, Germany) and used as a reference strain.

### 2.2. Identification of A. radioresistens Using VITEK2

For the identification of the bacterial species using the VITEK 2 system, biochemical profiles of the strains were recorded and compared with a stored database. Before starting the analysis, the bacteria were taken from the cryotubes and incubated on Columbia blood agar plates (bioMérieux, Nürtingen, Germany) overnight at 37 °C. Then, a single colony was picked and transferred to a new Columbia blood agar plate followed by an incubation step of 18 h at 37 °C according to the current EUCAST guidelines. Again, single colonies were picked and a McFarland standard of 0.5 was adjusted using 3 mL of a 0.45% sodium chloride solution and a VITEK DensiCheck Plus densitometer (bioMérieux, Nürtingen, Germany). For species identification, a GN card (bioMérieux, Nürtingen, Germany, designed for identification of Gram-negative bacteria) was applied [16].

### 2.3. Identification of A. radioresistens Using MALDI-TOF MS

All isolates were grown on Columbia blood agar plates (bioMérieux, Nürtingen, Germany) for 18 h at 37 °C. Then, a single colony was picked and plated on a 96-spot steel target (Bruker Daltonik, Bremen, Germany) and superimposed by α-Cyano-4-hydroxycinnamic acid (Bruker Daltonik, Bremen, Germany), which was used as an analytical matrix. The spectra were analyzed using a microflex MALDI-TOF MS system (Bruker Daltonik, Bremen, Germany) applying the flexControl software 3.1 (Bruker Daltonik, Bremen, Germany). This method has been described in more detail in a previous publication by our group [16]. For the MALDI-TOF MS experiments, calibration and positive control were performed with the BRUKER bacterial test standard (BTS).

### 2.4. Sequencing of the 16S rRNA and rpoB Gene of A. radioresistens

The 16S rRNA and *rpoB* were analyzed using Sanger sequencing. In both cases, the respective primers were used for both the preceding PCR and Sanger sequencing (Supplementary Table S1) [17,18]. Oligonucleotides were purchased from biomers.net (Ulm, Germany). Detailed descriptions of the PCR reactions are provided in Supplementary Tables S2 and S3. The PCR product was purified enzymatically using Exonuclease I and Shrimp alkaline phosphatase (New England Biolabs, Frankfurt am Main, Germany). Sanger sequencing was performed by SEQLAB (Sequence Laboratories Göttingen, Göttingen, Germany). Sequences obtained were analyzed using NCBI BLAST (Version 2.9.0+) [19]. In this study, the guidelines provided (Cut-Off: 98.7%) by Yarza et al. for species identification based on the 16S rRNA gene were applied [20,21]. In addition, the proposal for *rpoB*-based species identification (Cut-Off: 97.7%) published by Adekambi et al. was used [22]. In all PCR experiments, negative control using water was performed. A replication control was performed by size-controlled gel electrophoresis prior to sanger sequencing.

### 2.5. Identification Using Average Nucleotide Identity and Digital DNA-DNA Hybridization

To confirm the correct taxonomic assignment of the *A. radioresistens* strains included in this study, we performed Whole Genome Sequencing (WGS) using Illumina technology. Nextera XT DNA libraries (Nextera XT DNA Library Prep Kit, Illumina, San Diego, CA, USA) were generated from genomic DNA and sequenced on an Illumina NextSeq sequencer (Illumina, San Diego, CA, USA) followed by short-read genome assemblies using SPAdes 3.12 [23] (Center for Algorithmic Biothechnology, St. Petersburg, Russia). All genome sequences were deposited at NCBI GenBank, BioProject ID PRJNA224116, Acc. Nos. JAATOZ000000000-JAATPL000000000 and JANRFO000000000-JANRFV000000000 (Table 3). In silico analyses were performed using the EZ Biocloud ANI calculator (https://www.ezbiocloud.net/tools/ani, 22 June 2022) and the DSMZ type strain genome server (TYGS, https://tygs.dsmz.de, 29 May 2021) to determine digital DNA-DNA hybridization values [24,25]. For taxonomic comparisons, our isolates were compared to the type strain genome *A. radioresistens* NBRC 102413^T^ (GenBank Acc. Nos. AP019740-1). For the determination of a species affiliation of a strain, a cut-off of 94–96% is given for the ANI and a value of 70% for the dDDH [21].

## 3. Results

### 3.1. Patient Characteristics and Samples

Clinical data were only available for the 16 isolates from Dresden. All isolates except one were part of a polymicrobial community (Table 1). The samples were obtained from the lower limb (*n* = 7), the inguinal/gluteal region (*n* = 3), the abdomen (*n* = 1), and the genitourinary tract (*n* = 3). One isolate was derived from a bronchial secretion (*n* = 1). Another strain was found in blood culture (*n* = 1). However, in the latter case, *A. radioresistens* was accompanied by *Staphylococcus saprophyticus* (Table 1). Two of the sixteen strains (DSM 108820 and DSM 109007) were isolated from the same Intensive Care Unit (ICU) patient from different spots at different times. Ten patients were male, and six patients were female. The age of the patients ranged from 23 years to 99 years. The median age was 64 years. Further details are provided in Table 1. The five strains provided by other research groups were derived from human (*n* = 3) or veterinary samples (*n* = 2). The species information was confirmed by our verification. Details are provided in Table 1.

### 3.2. Identification Using the VITEK 2 System

Overall, 17 of 21 strains tested were identified as *A. radioresistens* showing excellent results (99%). Two strains (DSM 108820 and DSM 109999) were identified as *A. lwoffii* with an excellent identification result as well (99%). Two strains (DSM 109007 and K51-37) were indicated with “low discrimination”. However, in the case of DSM 109007, the Advanced Expert System (AES) of the VITEK 2 proposed A. *lwoffii* and *Moraxella* spp.

### 3.3. Identification Using MALDI-TOF MS

All strains were identified as *A. radioresistens* with a score of 2.0 or higher. This can be interpreted as secure identification at the species level [16]. A total of 15 of the 21 strains were identified with a score value of ≥2.3, which is considered a very secure identification of the species. Moreover, 6 of 21 strains were securely identified with a score between 2.0 and 2.3 [16].

### 3.4. Results from Sequencing of the 16S rRNA and rpoB Gene of A. radioresistens

All isolates were identified as *A. radioresistens* using sequencing of the 16S rRNA gene. In three isolates (DSM 108289, DSM 108820 and DSM 108297) the identity was slightly below the currently proposed cut-off value for species identification based on 16S rRNA sequencing. Results obtained from sequencing of *rpoB* revealed an excellent identification of *A. radioresistens* in all strains (Table 4).

### 3.5. Results Obtained from Average Nucleotide Identity and Digital DNA-DNA Hybridization

The cut-off-value for species identification using ANI is indicated with a similarity of 94–96% [21]. For the calculation of ANI, a web-based tool was used [25]. Sequences were compared with the draft genome sequence of the type strain (GenBank Acc. No. AP019740.1). The results for all strains included in this study revealed values higher than 98.18%. All strains were therefore identified and confirmed as *A. radioresistens*. The TYGS tool was used to calculate dDDH values [25]. The genomes of our strains were compared with the genome of the type strain NBRC 102413^T^ (AP019740.1), (which is identical to DSM 6976^T^). All values obtained were above 70%. However, since the genome data obtained in this study is not fully complete, the d4 formula was chosen for analysis. Nevertheless, taking the conclusions from Kolthoff et al. into consideration, one can state that all strains can be identified securely [14]. Detailed dDDH values are provided in Table 4.

## 4. Discussion

The present study aims to define the most suitable method that grants a reliable identification of the species *A. radioresistens* during routine diagnostics. This knowledge is an indispensable prerequisite for establishing a reference collection from clinical isolates, which will subsequently allow more detailed investigations on the pathogenicity of *A. radioresistens* and eventually helps to clarify the clinical significance of the species. To the best of our knowledge, the study presented here is the first, which solely focuses on the identification of *A. radioresistens* by diagnostic routine methods.

Our results show that the VITEK 2 system may lead to false identifications (4 out of 21 isolates were misidentified or could not be discriminated) and is therefore not the best choice to screen for *A. radioresistens* during routine diagnostics. Instead of the correct species *A. radioresistens*, phylogenetically related species such as *A. lwoffii* or *Moraxella* spp. were proposed by the VITEK 2 AES (Table 4, Supplementary Table S4). It is therefore very likely that misidentification is due to similar metabolic properties. However, the metabolic patterns of this species are well studied [27,28,29,30] and therefore a modification of the VITEK 2 GN card may lead to more reliable results.

In contrast to this, the sequencing of the 16S rRNA or the *rpoB* gene leads to more reliable identification results (Table 4). However, regarding the sequencing of the 16S rRNA gene it should be noted that we found values slightly below the currently defined cut-off for species identification in three isolates (DSM 108289, DSM 108820 and DSM 108297). Moreover, specially trained personnel and an elaborate laboratory infrastructure are needed to carry out these procedures. In addition, time-consuming manual analysis steps are often necessary, and the result is often only available after a few days. For this reason, not all diagnostic laboratories provide this kind of analysis. Therefore, the sequencing of both the 16S rRNA and *rpoB* is unsuitable for screening for *A. radioresistens* during routine diagnostics but could make an important contribution to verifying or falsifying an uncertain identification result. 

Furthermore, our results suggest that MALDI-TOF MS can identify *A. radioresistens* with high reliability and reproducibility (Table 4). Moreover, analyses are easy to handle, and the method is cost-effective. Another advantage is the fast provision of analysis results [31,32]. For these reasons, MALDI-TOF MS can be used as a high-throughput method and is therefore suitable for the targeted identification of *A. radioresistens* from clinical isolates. Therefore, MALDI-TOF MS might be the best approach to detect *A. radioresistens* strains during routine diagnostics for building a reference collection.

However, most of the studies that have investigated the suitability of MALDI-TOF MS for the identification of *Acinetobacter* spp. so far dealt with the clinically relevant species of the *A. calcoaceticus*–*A. baumannii* complex. This currently includes *A. baumannii*, *A. calcoaceticus*, *A. pittii*, *A. nosocomialis*, and the two recently added species *A. seifertii* and *A. lactucae* [33,34]. In some of these previous studies, *A. radioresistens* isolates were also investigated. However, it must be clearly stated that the number of strains examined here was confined. Li et al. or Ha et al. for example included only one and Hsueh et al. only a total of three [34,35,36]. Furthermore, despite intensive efforts, we were only able to obtain a limited number of isolates from our routine diagnostics. For these reasons, it is currently difficult to make a general statement on the suitability of MALDI TOF MS for the identification of *A. radioresistens* in routine microbiological diagnostics. Nonetheless, these results already indicated that *A. radioresistens* is reliably identified using MALDI-TOF MS, which is in accordance with the results demonstrated here. Furthermore, it must be emphasized, that neither of the previous studies used NGS-based methods such as dDDH as a reference method to verify the species as *A. radioresistens*.

It is desirable to incorporate more isolates in a future study in order to make these preliminary results more secure because our data are mostly derived from our laboratory. Furthermore, because genetic traits vary within one species, further strains of different origins and regions must be included in future studies to confirm our results presented here.

A major advantage of our study is the use of both ANI and digital DNA-DNA hybridization as analysis methods. The determined values in both strategies were higher than the cut-off values that were defined for each method. Therefore, the species *A. radioresistens* was confirmed by two different analytical strategies, which are currently regarded as the molecular gold standard of species identification [14,15,24].

Presently, *A. radioresistens* has been linked to human infections in only a very limited number of cases [5,6,7,8,9]. Most of the patients reported in those publications were found to have limitations of their immune system. Furthermore, most of the patients were at a more advanced age (Table 1). This is important insofar as aging is also associated with gradually increasing restrictions of the immune system [37]. Taking this fact into account, one could assume that our patients may be at an increased risk for acquiring an *A. radioresistens* infection. Nevertheless, it can be expected that more and more cases will be recognized with time because advanced methods of bacterial identification such as MALDI-TOF MS become increasingly widespread in diagnostic laboratories [31,32].

To sum up, our results indicate that MALDI-TOF MS may be the best method to detect *A. radioresistens* during routine diagnostics. This is also ensured by using both ANI and dDDH as appropriate analysis methods used in studies, which are aimed to compare different methods for bacterial identification.

## Figures and Tables

**Table 1:** Clinical data on the *A. radioresistens* strains from the strains collected in Dresden.

Strain	Sex	Age (Years)	Source of Isolation	Microbial Spectrum Detected	Underlying Disease	Year of Isolation
**DSM 108289**	m	55	Swab (foot)	Skin flora, *Staphylococcus aureus*	Diabetic foot syndrome	2013
**DSM 108290**	m	74	Swab (foot)	Skin flora, coagulase-negative *Staphylococcus* spp. (nfdp)	Diabetic foot syndrome	2014
**DSM 108291**	m	58	Urine (midstream)	Flora of anterior urethra, *Candida palmioleophila*	Urologic (n.g.)	2013
**DSM 108292**	m	89	Blood culture (peripheral venous)	*Staphylococcus saprophyticus*	Fever of unknown origin (n.g.)	2013
**DSM 108293**	f	23	Swab (vaginal)	Vaginal normal flora	Gynecological, control in pregnancy	2013
**DSM 108294**	m	46	Swab (foot)	Gram-positive anaerobic rods (nfdp), Gram-negative anaerobic rods (nfdp), *Citrobacter freundii*, *Staphylococcus aureus*, *Pseudomonas aeruginosa*	Neurological, diabetic foot syndrome	2013
**DSM 108295**	m	87	Urine (midstream)	Flora of anterior urethra	Urological (n.g.)	2013
**DSM 108296**	f	99	Swab (heel)	Skin flora, *Staphylococcus aureus*, *Gleimia europaea*	General surgery (n.g.)	2013
**DSM 108297**	m	47	Swab (ulcer lower leg)	Skin flora, anaerobic skin flora, *Enterobacter cloacae* cplx., *Pantoea* spp., *Bacillus cereus*	Neurological (n.g.)	2013
**DSM 108349**	f	52	Swab (abdomen)	Skin flora, *Staphylococcus aureus*, *Pseudomonas* spp.	Dermatological, Psoriasis vulgaris	2018
**DSM 108719**	m	73	Swab (lower leg)	Skin flora	Dialysis (n.g.)	2018
**DSM 108820**	f	61	Swab (gluteal)	-	Brain injury, hemiparesis, bronchitis	2020
**DSM 109007**	f	61	Bronchial secretion	Oral and pharygeal flora, *Candida albicans*	Brain injury, hemiparesis, bronchitis	2020
**DSM 109999**	f	59	Swab (inguinal)	Skin flora, *Staphylococcus aureus*, *Escherichia coli*	Tinea	2019
**DSM 112285**	m	67	Swab (lower lower leg)	*Staphylococcus aureus*, *Enterococcus faecalis*, anaerobic skin flora	Endocrinology, diabetic foot syndrome	2020
**DSM 112286**	m	67	Swab (inguinal)	*Pseudomonas stutzeri*, Gram-negative rods (nfdp)	Traumatic brain injury, brain oedema, pneumonia, dysphagia	2020

m = male; f = female; n.g. = not given; nfdp = no further differentiation possible.

**Table 2:** Strains included from other institutions.

Strain	Isolation Source	Reference
**K60-62**	Human blood culture, nfi	Karah et al., 2011, Provided by Ørjan Samuelsen [17]
**K51-37**	Human blood culture, nfi	Karah et al., 2011, Provided by Ørjan Samuelsen [17]
**LH 5**	Poultry feces	Crippen et al., 2020, Provided by Christine Szymanski [26]
**LH 6**	Poultry feces	Crippen et al., 2020, Provided by Christine Szymanski [26]
**R 866 BER**	Human skin or urinary tract, nfi	Poirel et al., 2008, Provided by Patrice Nordmann [10]

nfi = no further information provided.

**Table 3:** Genome data of all *A. radioresistens* strains sequenced within this study.

Strain	GenBank Acc. No.	Genome Size (Mbp)	No. Contigs	No. CDS	GC%	Seq. Method/Quality
**DSM 108289**	JAATOZ01	3.32882	78	3083	41.4	WGS/Contig
**DSM 108290**	JAATPA01	3.18361	65	2897	41.5	WGS/Contig
**DSM 108291**	JAATPB01	3.33435	83	3100	41.5	WGS/Contig
**DSM 108292**	JAATPC01	3.26389	77	3008	41.6	WGS/Contig
**DSM 108293**	JAATPD01	3.35282	95	3080	41.5	WGS/Contig
**DSM 108294**	JAATPE01	3.25912	59	3034	41.6	WGS/Contig
**DSM 108295**	JAATPF01	3.23626	86	2977	41.6	WGS/Contig
**DSM 108296**	JAATPG01	3.03875	42	2754	41.6	WGS/Contig
**DSM 108297**	JAATPH01	3.17131	71	2938	41.6	WGS/Contig
**DSM 108349**	JAATPI01	3.21774	91	2974	41.5	WGS/Contig
**DSM 108719**	JAATPJ01	3.21258	109	2979	41.8	WGS/Contig
**DSM 108820**	JAATPK01	3.21113	54	2955	41.6	WGS/Contig
**DSM 109007**	JAATPL01	3.46228	65	3211	41.2	WGS/Contig
**DSM 109999**	JANRFV00	3.18874	67	2942	41.6	WGS/Contig
**DSM 112285**	JANRFU00	3.26273	47	3012	41.5	WGS/Contig
**DSM 112286**	JANRFT00	3.32056	56	3114	41.5	WGS/Contig
**K60-62**	JANRFS00	3.23746	82	3026	41.5	WGS/Contig
**K51-37**	JANRFR00	3.30427	77	3080	41.5	WGS/Contig
**LH 6**	JANRFP00	3.03215	59	2776	41.7	WGS/Contig
**LH 5**	JANRFQ00	3.04738	51	2780	41.5	WGS/Contig
**R 866 BER**	JANRFO00	3.08218	65	2844	41.6	WGS/Contig

**Table 4:** Comparative overview of the applied diagnostic procedures.

Identification	Results Obtained by VITEK 2 ^1^	Results Obtained by MALDI-TOF MS ^2^	Results Obtained by Sequencing of the 16S rRNA Gene ^3^	Results Obtained by Sequencing of the *rpoB* Gene ^4^	Results Obtained by Calculating ANI ^5^	Results Obtained by Calculating dDDH ^6^
**DSM 108289**	*A. radioresistens* (99%)	*A. radioresistens* 2.29	*A. radioresistens* 98.00%	*A. radioresistens* 99.71%	98.46%	85.9%
**DSM 108290**	*A. radioresistens* (99%)	*A. radioresistens* 2.49	*A. radioresistens* 99.21%	*A. radioresistens* 99.11%	98.36%	85.8%
**DSM 108291**	*A. radioresistens* (99%)	*A. radioresistens* 2.44	*A. radioresistens* 99.33%	*A. radioresistens* 99.12%	98.34%	86.0%
**DSM 108292**	*A. radioresistens* (99%)	*A. radioresistens* 2.33	*A. radioresistens* 99.46%	*A. radioresistens* 98.77%	98.41%	86.2%
**DSM 108293**	*A. radioresistens* (99%)	*A. radioresistens* 2.31	*A. radioresistens* 99.13%	*A. radioresistens* 99.70%	98.44%	86.1%
**DSM 108294**	*A. radioresistens* (99%)	*A. radioresistens* 2.34	*A. radioresistens* 99.41%	*A. radioresistens* 99.71%	98.53%	86.8%
**DSM 108295**	*A. radioresistens* (99%)	*A. radioresistens* 2.44	*A. radioresistens* 99.02%	*A. radioresistens* 98.8%	98.33%	86.1%
**DSM 108296**	*A. radioresistens* (99%)	*A. radioresistens* 2.23	*A. radioresistens* 99.55%	*A. radioresistens* 98.47%	98.40%	86.1%
**DSM 108297**	*A. radioresistens* (99%)	*A. radioresistens* 2.24	*A. radioresistens* 97.82%	*A. radioresistens* 99.71 %	98.45%	86.4%
**DSM 108349**	*A. radioresistens* (99%)	*A. radioresistens* 2.49	*A. radioresistens* 99.76%	*A. radioresistens* 99.40%	98.31%	85.8%
**DSM 108719**	*A. radioresistens* (99%)	*A. radioresistens* 2.32	*A. radioresistens* 99.46%	*A. radioresistens* 99.12%	98.32%	84.7%
**DSM 108820**	*A. lwoffii* (99%)	*A. radioresistens* 2.49	*A. radioresistens* 98.03%	*A. radioresistens* 99.41%	98.39%	86%
**DSM 109007**	*Low discrimination* (*A. lwoffi*, *Moraxella* spp.)	*A. radioresistens* 2.14	*A. radioresistens* 99.34%	*A. radioresistens* 99.70%	98.38%	86.7%
**DSM 109999**	*A. lwoffii* (99%)	*A. radioresistens* 2.41	*A. radioresistens* 98.82%	*A. radioresistens* 99.70%	98.45%	86.1%
**DSM 112285**	*A. radioresistens* (99%)	*A. radioresistens* 2.45	*A. radioresistens* 99.29%	*A. radioresistens* 100.00%	98.35%	85.7%
**DSM 112286**	*A. radioresistens* (99%)	*A. radioresistens* 2.29	*A. radioresistens* 99.79%	*A. radioresistens* 99.14%	98.46%	85.7%
**K 50-62**	*A. radioresistens* (99%)	*A. radioresistens* 2.39	*A. radioresistens* 99.51%	*A. radioresistens* 100.00%	98.39%	84.5%
**K 51-37**	*Low discrimination* (*A. lwoffii*, *A. radioresistens*)	*A. radioresistens* 2.45	*A. radioresistens* 99.65%	*A. radioresistens* 100.00%	98.36%	85.4%
**LH 5**	*A. radioresistens* (99%)	*A. radioresistens* 2.09	*A. radioresistens* 99.51%	*A. radioresistens* 98.54%	98.33%	84.9%
**LH 6**	*A. radioresistens* (99%)	*A. radioresistens* 2.33	*A. radioresistens* 99.54%	*A. radioresistens* 99.12%	98.34%	86.2%
**R 866 BER**	*A. radioresistens* (99%)	*A. radioresistens* 2.30	*A. radioresistens* 99.89%	*A. radioresistens* 98.82%	98.46%	85.9%

^1^—Identification result; ^2^—Highest score, (BRUKER Biotyper score with values between 0.0 and 3.0, 1.7–1.99—low confidence identification, >2.00—high confidence identification, <1.7—no reliable identification) [16]; ^3^—Perc. identity with NCBI 16S ribosomal RNA sequences database; ^4^—Perc. identity in % (Reference from NCBI); ^5^—Average nucleotide identity compared with NBRC 102413^T^ (AP019740.1) in %; ^6^—dDDH (d4, in %) compared with DSM 6976^T^.

## Supplementary Materials

The following Supplementary Materials are available online at https://www.mdpi.com/article/10.3390/microorganisms10091767/s1, Table S1: PCR Primer sequences, Table S2: PCR cycling protocol *rpoB*, Table S3: PCR protocol 16S *rRNA*, Table S4: Results obtained from VITEK 2.
