# Supplementary material for: Comparative Study of Different Diagnostic Routine Methods for the Identification of Acinetobacter radioresistens
Source: Microorganisms. 2022 Aug 31;10(9):1767. doi: 10.3390/microorganisms10091767 (PMC9503985; doi:10.3390/microorganisms10091767)
Supplement: Supplementary file 1 [file microorganisms-10-01767-s001.zip › Supplementary Table S1 .pdf]

Supplementary Table S1: Primer sequences used in this study

Name	Forward (f)/Reverse (r) Primer	Primer sequence	Reference
16s-DNA-F	f	3`-AGAGTTTGATCMTGGCTCAG-5`	[17]
16s DNA-R	r	3`-ACGGHTACCTTGTTACGACTT-5`	[17]
Ac696F	f	3`-AGAGTTTGATCMTGGCTCAG-5`	[18]
Ac109R	r	3`-ACGGHTACCTTGTTACGACTT-5`	[18]
