# Supplementary material for: Comparative Study of Different Diagnostic Routine Methods for the Identification of Acinetobacter radioresistens
Source: Microorganisms. 2022 Aug 31;10(9):1767. doi: 10.3390/microorganisms10091767 (PMC9503985; doi:10.3390/microorganisms10091767)
Supplement: Supplementary file 1 [file microorganisms-10-01767-s001.zip › Supplementary Table S2.pdf]

Supplementary Table S2: PCR Cycling Protocols

Temprature	Time	Comment
**94°C**	2 min	Initial denaturation
**94°C**	30 sec	Cycle: denaturation (35 cycles)
**52°C**	30 sec.	Cycle: annealing (35 cycles)
**72°C**	1 min	Cycle: elongation (35 cycles)
**72°C**	7 min	Termination
**4°C**	∞	Hold

Protocol for *rpoB* gene sequencing
