# Supplementary material for: Comparative Study of Different Diagnostic Routine Methods for the Identification of Acinetobacter radioresistens
Source: Microorganisms. 2022 Aug 31;10(9):1767. doi: 10.3390/microorganisms10091767 (PMC9503985; doi:10.3390/microorganisms10091767)
Supplement: Supplementary file 1 [file microorganisms-10-01767-s001.zip › Supplementary Table S3.pdf]

Supplementary Table S3

**Temprature**	**Time**	**Comment**	
**95°C**	5 min	Initial denaturation	
**95°C**	1 min	Cycle: denaturation	
**50°C**	1 min.	Cycle: annealing	35 cycles
**72°C**	90 sec	Cycle: elongation	
**72°C**	7 min	Termination	
**4°C**	∞	Hold	

Protocol for *16s rDNA* Gene sequencing
