# Supplementary material for: Comparative Study of Different Diagnostic Routine Methods for the Identification of Acinetobacter radioresistens
Source: Microorganisms. 2022 Aug 31;10(9):1767. doi: 10.3390/microorganisms10091767 (PMC9503985; doi:10.3390/microorganisms10091767)
Supplement: Supplementary file 1 [file microorganisms-10-01767-s001.zip › Supplementary Table S4.pdf]

Supplementary Table S4: Results obtained from VITEK 2

**Strain**	**ILATk**	**TyrA**	**MNT**	**ILATa**	**Indentification:**
DSM 108719	+	+	+	-	Acinetobacter radioresistens (99%)
DSM 108296	+	+	+	-	Acinetobacter radioresistens (99%)
DSM 108249	+	+	+	-	Acinetobacter radioresistens (99%)
DSM 108290	+	+	+	-	Acinetobacter radioresistens (99%)
DSM 108297	+	+	+	-	Acinetobacter radioresistens (99%)
DSM 108291	+	+	+	-	Acinetobacter radioresistens (99%)
DSM 108289	+	+	+	-	Acinetobacter radioresistens (99%)
DSM 108293	+	+	+	-	Acinetobacter radioresistens (99%)
DSM 108295	+	+	+	+	Acinetobacter radioresistens (99%)
DSM 108292	+	+	+	-	Acinetobacter radioresistens (99%)
DSM 108294	+	+	+	+	Acinetobacter radioresistens (99%)
DSM 108820	+	+	-	-	Acinetobacter lwoffi (99%)
DSM 109007	+	+	-	-	Low discriminatiion
DSM 109999	+	+	-	+	Acinetobacter lwoffii (99%)
DSM 108297	+	+	+	+	Acinetobacter radioresistens (99%)
DSM 108349	+	+	+	-	Acinetobacter radioresistens (99%)
K 50-62	+	+	+	-	Acinetobacter radioresistens (99%)
K 51-37	+	+	-	-	Low discriminatiion

LH 5	+	+	+	-	Acinetobacter radioresistens (99%)
LH 6	+	+	+	-	Acinetobacter radioresistens (99%)
R 866 BER	+	+	+	-	Acinetobacter radioresistens (99%)

+ Positive result, - negative result, substances which have been tested, but were negative in all isolates are not shown
